# Emotion-induced brain activation across the menstrual cycle in individuals with premenstrual dysphoric disorder and associations to serum levels of progesterone-derived neurosteroids

**DOI:** 10.1038/s41398-023-02424-3

**Published:** 2023-04-14

**Authors:** Louise Stiernman, Manon Dubol, Erika Comasco, Inger Sundström-Poromaa, Carl-Johan Boraxbekk, Maja Johansson, Marie Bixo

**Affiliations:** 1 Department of Clinical Sciences, Umeå University, Umeå, Sweden; 2 Department of Women’s and Children’s Health, Science for Life Laboratory, Uppsala University, Uppsala, Sweden; 3 Department of Women’s and Children’s Health, Uppsala University, Uppsala, Sweden; 4 Department of Radiation Sciences, Diagnostic Radiology, Umeå University, Umeå, Sweden; 5 Institute for Clinical Medicine, Faculty of Medical and Health Sciences, University of Copenhagen, Copenhagen, Denmark; 6 Danish Research Centre for Magnetic Resonance (DRCMR), Centre for Functional and Diagnostic Imaging and Research, Copenhagen University Hospital - Amager and Hvidovre, Copenhagen, Denmark; 7 Umeå Center for Functional Brain Imaging (UFBI), Umeå University, Umeå, Sweden; 8 Institute of Sports Medicine Copenhagen (ISMC) and Department of Neurology, Copenhagen University Hospital Bispebjerg, Copenhagen, Denmark

**Keywords:** Neuroscience, Pathogenesis, Diseases

## Abstract

Premenstrual dysphoric disorder (PMDD) is a debilitating disorder characterized by severe mood symptoms in the luteal phase of the menstrual cycle. PMDD symptoms are hypothesized to be linked to an altered sensitivity to normal luteal phase levels of allopregnanolone (ALLO), a GABA_A_-modulating progesterone metabolite. Moreover, the endogenous 3*β*-epimer of ALLO, isoallopregnanolone (ISO), has been shown to alleviate PMDD symptoms through its selective and dose-dependent antagonism of the ALLO effect. There is preliminary evidence showing altered recruitment of brain regions during emotion processing in PMDD, but whether this is associated to serum levels of ALLO, ISO or their relative concentration is unknown. In the present study, subjects with PMDD and asymptomatic controls underwent functional magnetic resonance imaging (fMRI) in the mid-follicular and the late-luteal phase of the menstrual cycle. Brain responses to emotional stimuli were investigated and related to serum levels of ovarian steroids, the neurosteroids ALLO, ISO, and their ratio ISO/ALLO. Participants with PMDD exhibited greater activity in brain regions which are part of emotion-processing networks during the late-luteal phase of the menstrual cycle. Furthermore, activity in key regions of emotion processing networks - the parahippocampal gyrus and amygdala - was differentially associated to the ratio of ISO/ALLO levels in PMDD subjects and controls. Specifically, a positive relationship between ISO/ALLO levels and brain activity was found in PMDD subjects, while the opposite was observed in controls. In conclusion, individuals with PMDD show altered emotion-induced brain responses in the late-luteal phase of the menstrual cycle which may be related to an abnormal response to physiological levels of GABA_A_-active neurosteroids.

## Introduction

The prevalence of premenstrual dysphoric disorder (PMDD) has been estimated at 3–5% during childbearing ages [1, 2]. The condition significantly impacts the quality of life [3, 4] and is characterized by mood symptoms - irritability, depressed mood, anxiety and emotional lability - which are present only in the premenstrual (luteal) phase of the menstrual cycle.

PMDD symptoms are relieved when ovarian hormone production is suppressed [5], or kept at constant and low levels [6], and can be elicited by progesterone or estradiol administration [7–9]. A growing body of evidence suggests that the potent, centrally active *γ*-aminobutyric acid type A (GABA_A_) receptor-modulating metabolite of progesterone, allopregnanolone (ALLO), plays an important role in PMDD symptomatology [10, 11]. Serum levels of ALLO closely follow those of circulating progesterone across the menstrual cycle with an offset of 2–3 days [12–14]. In common with other GABA_A_-receptor agonists (such as benzodiazepines and barbiturates), ALLO has anesthetic, antiepileptic and anxiolytic properties in animals, and is sedative when given at supra-physiological doses to humans [15, 16]. Individuals with PMDD, however, seem to have an altered pharmacodynamic response to ALLO across the menstrual cycle compared with controls [14]. For instance, individuals with PMDD appear to be more sensitive to ALLO (as measured by changes in saccadic eye velocity) in the luteal phase compared to the follicular phase, which is opposite to the pattern seen in asymptomatic controls [15]. This would suggest an inability in PMDD to develop tolerance to increased levels of neurosteroids active at the GABA_A_-receptor in the luteal phase. Positive treatment effects have been shown in response to 5α-reductase inhibitors, which block the conversion of progesterone to ALLO [17]. Moreover, the therapeutic effect of another endogenous neurosteroid, isoallopregnanolone (ISO), has been investigated in PMDD with promising results [18, 19]. ISO is also metabolized from progesterone and its levels are increased during the luteal phase of the menstrual cycle [20]. ISO directly antagonizes ALLO’s effect on the GABA_A_ receptor in a dose-dependent manner but does not on its own inhibit GABA-evoked currents [20–23]. Moreover, the action of ISO seems to be specific to ALLO as it does not antagonize the effect of other GABA_A_-receptor agonists such as benzodiazepines or barbiturates [24, 25]. In healthy individuals, ISO given intravenously noticeably antagonizes ALLO’s pharmacodynamic effects at serum concentrations half of that of ALLO [23], indicating that the relative concentration of the two steroids is significant with regards to GABA_A_-receptor activation. Interestingly, epimerization between ISO and ALLO has been suggested to be a biological mechanism by which GABA_A_ receptor tone is modulated, and an imbalance between ALLO and ISO levels has been implicated in a number of psychopathologies, including major depression [26, 27]. While it is currently unknown whether an imbalance between ISO and ALLO concentrations contributes to the pathophysiology of PMDD, there are indications that altering the balance between the two steroids may be beneficial. Indeed, apart from the efficacy of ISO as treatment for PMDD [19], SSRIs, which are currently the first line of treatment for the disorder, have been shown to alter ALLO and ISO levels in plasma and cerebrospinal fluid [28, 29]. Further research is warranted to determine the impact the relative concentration of ISO and ALLO have on GABA_A_-receptor activity and brain function in individuals with PMDD.

Functional resonance magnetic imaging (fMRI) studies of PMDD have provided evidence for dysregulation in emotion processing networks in subjects with PMDD [30]. Among the most robust findings are heightened amygdala and insula responses, along with decreased responses in the anterior cingulate cortex (ACC), which have been observed in the luteal phase during emotion-processing tasks in subjects with PMDD [31–34]. These regions are key parts of a hypothesized emotion processing network [35], whereby increased bottom-up activation of important hubs of the salience network (amygdala, insula) is accompanied by blunted responses in frontocingulate cortical regions (ACC, medial prefrontal cortex (mPFC), dorsolateral prefrontal cortex (dlPFC), leading to aberrant functional integration and connectivity within the salience network [36]. Few studies have explored how brain function is related to ovarian hormone levels in individuals with PMDD. One study found that phase-related changes in amygdala response correlated positively to progesterone in subjects with PMDD [32]. Another study reported positive correlations between progesterone and activation in the dorsolateral prefrontal cortex, as well as a positive relationship between estradiol and medial prefrontal cortex recruitment in the luteal phase in PMDD [37]. No study to date has investigated the relationship between endogenous GABA_A_-active neurosteroids and emotion-induced brain activity in PMDD.

The present study aimed to 1) investigate brain activity in response to emotional stimuli in subjects with PMDD and controls across the menstrual cycle, and 2) explore whether serum levels of ovarian hormones and GABA_A_-active neurosteroids are differently associated to emotion-induced brain activity in subjects with PMDD and controls. Here, we included the ratio variable ISO/ALLO in order to specifically investigate group differences in the relationship between the relative concentration of the steroids and brain activity. In line with previous fMRI findings in PMDD, we expected altered recruitment of brain regions involved in emotion processing, including the amygdala, during the symptomatic late-luteal phase. We also hypothesized that neurosteroid levels, and especially the ratio ISO/ALLO, would be differentially associated to brain activity in key regions of emotion-processing networks.

## Materials and methods

### Subjects

The study sample consisted of 31 participants with PMDD and 31 asymptomatic controls recruited by advertisement in local newspapers, on a student website for clinical trials, via social media platforms, and by posters at out-patient clinics. Subjects were eligible for inclusion if they were aged 18–45 years, had regular menstrual cycles (25–31 days), used non-hormonal contraception, fulfilled PMDD diagnostic criteria according to the Diagnostic and Statistical Manual of Mental Disorders, 5^th^ Ed (DSM-V) (PMDD group), were otherwise essentially healthy (PMDD and control group), and provided oral and written informed consent. Current use of steroid hormones, psychotropic or anti-depressant medication, significant somatic or psychiatric conditions, drug or alcohol abuse, pregnancy and contraindications for MRI were grounds for exclusion. Prior to entering the study, we required wash-out periods of three months for psychotropic drugs (such as selective serotonin re-uptake inhibitors and benzodiazepines) or alternative medicines with potential effects on mood, and one month for hormonal contraceptives. Potential participants were screened for psychiatric conditions (past and current) by the investigator using the Mini International Neuropsychiatric Interview questionnaire [38]. Psychosis, bipolar disorders, all anxiety disorders and alcohol or substance abuse etc. detected by the Mini were considered to be significant psychiatric conditions and thereby reasons for exclusion. Due to their high prevalence, moderate symptoms of an eating disorder, as well as past major depressive episodes were allowed, if they had been in remission for more than two years. All participants completed prospective daily ratings of PMDD symptoms for a minimum of two menstrual cycles using the Daily Record of Severity of Problems (DRSP), a validated diagnostic tool for PMDD [39]. The DRSP was implemented via an ad-hoc web platform and daily text reminders were sent encouraging participants to log their symptoms. The study was approved by the Regional Ethical Review Board in Umeå (2016-111-31 M, 2017-266-32 M).

### PMDD diagnosis

PMDD was diagnosed using the algorithm developed by Endicott *et al*. [39]. The criteria were as follows: 1) no average daily symptom score greater than 3 (“mild”) during the mid-follicular phase (days +6 to +10 after the onset of menses), 2) during the late-luteal phase (days −5 to −1 prior to the onset of menses), at least two days with ratings ≥4 (“moderate”) on a minimum of one “core” mood symptom (depressed mood, anxiety, affective lability, irritability) and on at least five symptoms overall. To specifically select a group of participants with severe PMDD we added the criterion, 3) symptoms in the late-luteal phase interfered with daily functioning, which was defined as ratings of ≥4 for two days on at least one impairment item (interference with work/school, social activities, relationships). A diagnosis of PMDD was given if the above criteria were met for two consecutive menstrual cycles. Participants included in the control group had to be asymptomatic across the entire menstrual cycle, i.e. no mean ratings >3 during either the mid-follicular phase or the late-luteal phase.

### Study design

Participants were scanned once in the asymptomatic mid-follicular phase (menstrual cycle day +5 to +11), and once in the late-luteal phase (menstrual cycle day −8 to −1). Late-luteal phase testing was planned to coincide with the peak in PMDD symptom severity [4]. Prior to each scanning session, a blood sample was drawn, and serum was frozen at −80^o^ within 30 min from collection for further analysis of ovarian hormones and neurosteroids. Ovulation was confirmed if serum progesterone concentrations fell within 2 standard deviations of the standard curve for progesterone for the corresponding luteal phase day [14]. To avoid test order effects, the menstrual cycle phase during which participants underwent their first scanning session was counterbalanced within each group: 44.4% of controls and 51.7% of PMDD subjects underwent their first scan in the mid-follicular phase.

### Steroid analyses

Serum concentrations of ALLO and ISO were analyzed by LabLytica, Uppsala, Sweden. In a first step, serum samples were extracted using liquid-liquid extraction in a hexane/ether solvent phase. They were then derivatized using 3-aminooxypropyl (trimethyl) ammonium bromide and quantified using ultra-high performance liquid chromatography-mass spectrometry (UPLC-MS/MS). The samples were compared against a freshly prepared calibration curve in surrogate matrix (water) to determine their concentrations. The limit of quantification (LLOQ) for ALLO was 0.2 nM and for ISO 0.1 nM. Serum concentrations of progesterone and estradiol were analyzed by the central hospital laboratory at Norrlands University Hospital, Umeå, Sweden. Analyses were done using Elecsys^®^ Gen III immunoassays for progesterone and estradiol separately. Samples were incubated with progesterone- or estradiol-specific biotinylated antibodies, and thereafter, streptavidin-coated microparticles were added to each mix together with a rutenium complex marked derivate for each steroid, forming antibody hapten complexes. Quantification of steroids were done with chemiluminescence and samples were compared against a device-specific calibration curve to determine their concentrations. The detection limit for progesterone was 0.05 ng/ml and for estradiol 5 pg/ml.

### Experimental paradigm

The emotional discrimination task used in this study has been previously described [40]. Participants were presented with Ekman faces displaying expressions of anger or fear (emotion task) and vertical or horizontal ellipses (sensorimotor control task). Participants were instructed to select one of two images matching the emotion or orientation of a target image by pressing a button with the right index finger. Emotion and sensorimotor control task trials were presented in blocks of six, in which stimuli were presented for 4 s, interspaced with a fixation cross (2 s for the sensorimotor control task and a randomly selected duration of 2, 4 or 6 s for the emotion task). Emotional content and sex of the individuals depicted were balanced across trials, as was the orientation of shapes. The entire paradigm consisted of four blocks of faces (24 trials) and five blocks of shapes (30 trials). Accuracy and reaction times were registered for each trial.

### Image acquisition

Magnetic resonance [] images were acquired using a 3.0 T Discovery MR750 (General Electric, Madison, WI, USA) scanner available through the Umeå Center for Functional Brain Imaging (UFBI). The scanner was equipped with a 32-channel head coil. The stimulus presentation software E-prime (Psychology Software Tools, Sharpsburg, PA, USA) was used for paradigm handling and viewed through a tilted mirror attached to the head coil. fMRI images were acquired with a gradient echo planar imaging sequence [37 transaxial slices; thickness, 3.4 mm; gap; 0.5 mm, repetition time (TR), 2000 ms; echo time (TE), 30 ms; flip angle, 80°; field of view, 25 × 25 cm; 200 volumes; duration, 07:00 min]. High-resolution T1-weighted structural images were collected with a 3D fast spoiled gradient echo sequence (176 transaxial slices; thickness, 1 mm; TR, 8.2 ms; TE, 3.2 ms; flip angle, 12°; field of view, 25 × 25 cm; duration, 08:11 min). A field map was acquired prior to the fMRI images and used for controlling for magnetic field (B0) inhomogeneities [46 transaxial slices; thickness; 4 mm; gap; 0 mm; repetition time (TR), 800 ms; flip angle, 10°; field of view, 25.6 × 25.6 cm; duration, 01:05 min]. All sequences were acquired in the A/P (anterior-to-posterior) frequency-encoding direction.

### fMRI data preprocessing and analysis

Image processing was conducted using the Oxford Centre for Functional Magnetic Resonance Imaging of the Brain (FMRIB)’s Software Library (FSL), version 6.00 [41]. Preprocessing steps included motion correction (reference image = middle volume), correction for B0 inhomogeneities, slice timing correction, and spatial smoothing with a 5-mm full-width at half maximum (FWHM) Gaussian kernel. A rigid body registration with FMRIB’s Linear Image Registration Tool (FLIRT) [42, 43], 6 degrees of freedom (DOF), was used to co-register functional images to individual structural T1 images. Spatial normalization into Montreal Neurological Institute (MNI) space was performed by applying an initial registration with FLIRT, 12 DOF affine transformations, followed by a non-linear transformation using FMRIB’s Non-linear Image Registration Tool (FNIRT) [44] with a warp-resolution of 8 mm, and resulting in 2 × 2 × 2 mm^3^ voxels.

First-level temporal modelling within a general linear model (GLM) framework was performed with FSL Expert Analysis Tool (FEAT) to generate single subject 3D maps of parameter estimates the contrast of interest [Faces > Shapes]. Design matrices were convolved with the default gamma hemodynamic response (HRF) function. Six motion parameters estimated from the spatial realignment were added to the model as covariates of no interest, and frames corrupted by large movements detected by FSL’s Motion Outliers tool (default metric = refrms) were removed from the analyses. Outliers were defined as falling outside the boxplot cut-off of 75th percentile + 1.5 x interquartile range. A high-pass filter (cut-off = 90 s) was applied to attenuate the lowest frequency components (linear scanner drift).

### Statistical analyses

#### Group differences in emotion-induced brain activity in the amygdala and at the whole brain level

FSL’s non-parametric permutation testing Randomise tool [45] was used for statistical inference. Voxel-wise analyses were conducted both with a small volume correction (SVC) for the right and left amygdala and at the whole-brain level. The region-of-interest [46] mask for the left and right amygdalae was defined using the Harvard-Oxford Subcortical Structural Atlas (thresholded at 80% probability). In order to assess group x phase interactions for the contrast of interest [Faces > Shapes] without violating the assumption of exchangeability relied on by permutation tests, individual differences between follicular and luteal scans were first computed for each subject before testing for group differences using an unpaired two-sided t-test. Separate paired and unpaired two-sided t-tests were then conducted to detect effects of the menstrual cycle phase within each group, and group differences within each menstrual cycle phase, respectively. Additional analyses assessing the influence of psychiatric history on the findings were conducted, as this variable tended to differ between groups (see Table 1).

**Table 1:** Baseline characteristics of participants with PMDD and controls.

	PMDD (*N* = 29) Mean (SD) or N (%)		Controls (*N* = 27) Mean (SD) or *N* (%)	
Demographics				
Age (years)	28.5 (6.1)		28.3 (5.7)	
BMI	23.7 (3.2)		24.4 (4.1)	
Menstrual cycle length (days)	27.7 (1.9)		28.6 (1.9)	
Psychiatric history	9 (31.0)		3 (11.1)	
*Depression*	8 (27.6)		3 (11.1)	
*Eating disorder*	1 (3.45)		0	
Parous	9 (31.0)		7 (25.9)	
DRSP ratings	*Mid-follicular*	*Late-luteal*	*Mid-follicular*	*Late-luteal*
Total symptom score	25.5 (3.5)	56.7 (14.3)	24.2 (3.3)	24.4 (3.0)^a^
Depression score	3.5 (0.6)	8.3 (2.8)	3.4 (0.7)	3.5 (0.6)^a^
Anxiety score	1.2 (0.3)	2.8 (1.0)	1.1 (0.2)	1.1 (0.2)^a^
Emotion lability score	2.3 (0.4)	9.3 (3.1)	2.2 (0.4)	3.4 (0.4)^a^
Irritability score	2.4 (0.4)	5.3 (2.2)	2.4 (0.6)	2.9 (0.4)^a^

Steroids	*Mid-follicular* Mean (IQR) or *N* (%)	*Late-luteal* Mean (IQR) or *N* (%)	*Mid-follicular* Mean (IQR) or *N* (%)	*Late-luteal* Mean (IQR) or *N* (%)
Test day	+8.0 (1.9)	−4.4 (2.0)	+7.7 (1.4)	−3.9 (1.7)
Progesterone (nmol/L)	0.6 (0.5)	23.8 (17.5)	0.7 (0.5)	23.4 (20.4)
Estradiol (pmol/L)	309 (201)	424 (238)	263 (162)	432 (156)
ALLO (nmol/L)	0.337 (0.098)	2.110 (1.420)	0.419 (0.247)	2.208 (1.060)
*Missing*	6 (20.7)	0	3 (11.1)	2 (7.4)
ISO (nmol/L)	0.127 (0.020)	0.742 (0.484)	0.137 (0.044)	0.844 (0.652)
*Missing*^†^	21 (72.4)	0	16 (59.3)	3 (11.1)
ISO/ALLO (nmol/L)	0.367 (0.093)	0.349 (0.108)	0.271 (0.079)	0.358 (0.153)
*Missing*	21 (72.4)	0	16 (59.3)	3 (11.1)

Total symptom scores are the mean summed ratings for all 21 symptom items of the DRSP scale (minimum=21, maximum=126) over days +5 to +11 for the mid-follicular phase, and days −8 to −1 for the late-luteal phase. The depression scores include the DRSP items “depressed”, “hopeless” and “guilty” (minimum=3, maximum=18); anxiety scores correspond to the item “anxious” (minimum=1; maximum=6); emotion lability scores include the items “mood swings” and “easily hurt” (minimum=2; maximum=12); and irritability scores include the items “irritable” and “conflicts” (minimum=2, maximum=12). Differences between groups were assessed using Mann-Whitney U-tests for continuous variables, and Fisher’s exact tests for categorical variables.

^a^Significant group difference at *p* < 0.05. Abbreviations: ALLO, Allopregnanolone; BMI, body mass index; DRSP, Daily Record of Severity of Problems; IQR, Interquartile Range; ISO, Isoallopregnanolone; PMDD, Premenstrual Dysphoric Disorder; SD, Standard Deviation. ^†^The large proportion of missing values for serum ISO was due to the lower detection limit of the method (see Materials and Methods, Steroid analyses).

#### Group differences in associations between emotion-induced brain activity and serum steroid levels

To test whether the linear relationship between brain activity and serum steroid levels differed between subjects with PMDD and controls, a two-group with continuous covariate interaction analysis was conducted voxel-wise using Randomise. Analyses were restricted to voxels within a mask combining clusters showing increased recruitment during task [Faces>Shapes] for both the PMDD and control group across the menstrual cycle (Table S1). Steroid covariates were mean centered across all subjects and split into two separate regressors according to group before being included in the model, allowing for the detection of group differences in slope between the dependent variable (brain activity) and steroid levels. In addition, the ratio variable ISO/ALLO, was log-transformed prior to being input into the model in order to avoid problems related to the asymmetry of ratios [47]. Group-by-steroid level interaction effects on brain activity were investigated for progesterone, estradiol and ALLO in both menstrual cycle phases. Analyses of interaction effects for ISO and ISO/ALLO were restricted to the late-luteal phase due to the high number of values below LLOQ in the mid-follicular phase (72.4% in the PMDD group and 59.3% in controls).

#### Associations between emotion-induced brain activity and symptom severity in subjects with PMDD

In order to evaluate whether significant associations existed between brain activity measures and symptom severity, the linear relationship between brain activity and DRSP scores was tested voxel-wise using Randomise within a mask combining brain regions exhibiting significant task-related activation [Faces>Shapes] in subjects with PMDD during the late-luteal phase (Table S2). Specifically, the total 21 symptom DRSP score and core PMDD symptoms (depression, irritability, affective lability and anxiety) DRSP scores were investigated. DRSP scores were averaged over days −8 to −1 to reflect the late-luteal phase scanning window. DRSP scores were derived from ratings recorded during the screening period for the study.

For all permutation testing using Randomise, a total of 5000 permutations was performed to build up null distributions to test against. Results were considered significant at *p* < 0.05 Family Wise Error (FWE) corrected using the threshold-free cluster enhancement (TFCE) method [48]. Trend-level results were defined as 0.05 < *p* < 0.1 for FWE-corrected statistics. We report t-statistics and cluster results (anatomical location, location in functional networks according to Yeo et al. [49], size, and local maximum).

## Results

### Participant characteristics

Demographic and endocrine characteristics of the subjects included in the study are presented in Table 1. Due to withdrawal of consent (*N* = 2), anovulation (*N* = 3) and screening failure (*N* = 1), a total of 6 participants (2 PMDD, 4 controls) were excluded from the analyses, which thus included 29 subjects with PMDD and 27 controls.

Subjects with PMDD did not differ from controls in terms of age, body mass index, menstrual cycle length or parity. Participants with PMDD tended to have a higher burden of prior psychiatric disease than controls (*p* = 0.10). All participants included in the analyses showed typical increases in concentrations of progesterone and neurosteroids from the mid-follicular to the late-luteal phase. No group differences in steroid levels were apparent across the menstrual cycle. Group differences in ISO and ISO/ALLO levels in the mid-follicular phase were not computed as ISO levels fell below the LLOQ for most participants (72.4% of PMDD subjects and 59.3% of controls).

### Emotion discrimination task performance

**Fig. 1:**
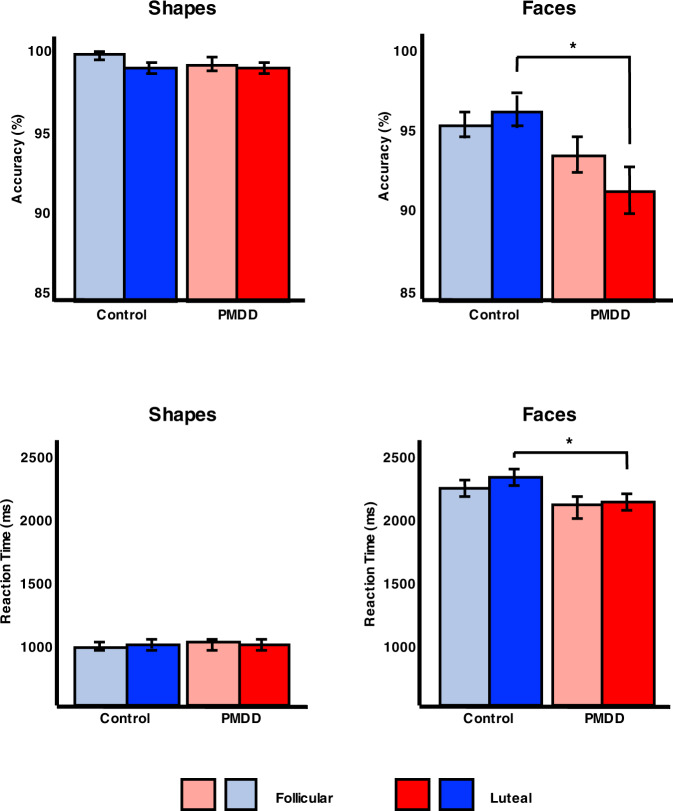
Emotional task performance in subjects with PMDD (*N* = 29) and controls (*N* = 27). Subjects with PMDD showed lower accuracy and reaction times in the face-matching condition compared with controls during the late-luteal phase. *p < 0.05. Error bars indicate 1 standard error of the mean. PMDD, premenstrual dysphoric disorder.

All participants showed high accuracy both when matching faces and shapes (>90% correct answers) across the menstrual cycle (Fig. 1, Table S3). In the late-luteal phase, subjects with PMDD were less accurate (*p* = 0.006) but faster (*p* = 0.046) compared with controls when matching faces. No differences in response accuracy or reaction time were found between groups in the shape-matching condition.

### Functional imaging results

#### Main effect of task

Significant task-related activations for the entire sample (both groups and sessions) at the whole-brain level for the Faces>Shapes contrast were found in regions previously described as constituents of emotional face processing networks [40, 50, 51] (Table S1).

#### Greater brain activity in the late-luteal phase in subjects with PMDD: amygdala and whole-brain results

**Fig. 2:**
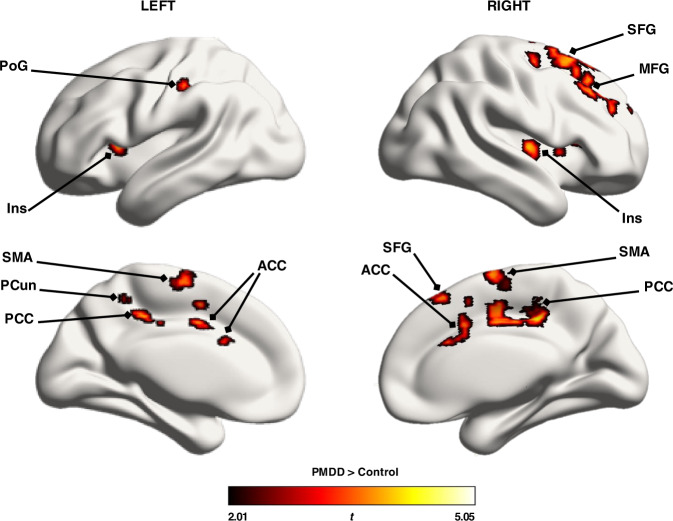
Whole-brain group comparison between subjects with PMDD (*N* = 29) and controls (*N* = 27) of emotion-induced brain activity during the late-luteal phase of the menstrual cycle (*p*_FWE_ < 0.05, TFCE). Surface representation of significant clusters showing increased brain activity during task [Faces>Shapes contrast] in subjects with PMDD, compared with controls, during the late-luteal phase across the whole brain (*p*_FWE_ < 0.05, TFCE). ACC Anterior Cingulate Cortex, FWE Family Wise Error correction, Ins Insula, MFG Medial Frontal Gyrus, PCC Posterior Cingulate Cortex, PCun Precuneus, PoG Postcentral Gyrus, PMDD Premenstrual Dysphoric Disorder, SFG Superior Frontal Gyrus, SMA Supplementary Motor Area, TFCE Threshold-Free Cluster Enhancement.

Whole-brain analyses revealed positive group x phase interactions in the right medial frontal gyrus (MFG) and right superior frontal gyrus (SFG) at trend-level (*p*_FWE_ < 0.10, TFCE) (Figure S1, Table S4). Brain activity was increased in these regions in the late-luteal phase compared to the mid-follicular phase in subjects with PMDD, while the opposite was seen in controls. Furthermore, group comparisons in the late-luteal phase revealed greater activations in subjects with PMDD compared to controls in areas of the ventral attention network: bilateral posterior cingulate cortices (PCC), left ACC, left precuneus, and bilateral insula; in regions belonging to the default mode network: right MFG and right SFG; as well as in the left supplementary motor area (SMA), left postcentral gyrus, dorsal striatum, thalamus and cerebellum (*p*_FWE_ < 0.05, TFCE) (Fig. 2, Table S5). No significant group effects were observed in the mid-follicular phase, nor were significant effects of phase apparent in either group. The results were not significantly influenced by psychiatric history.

Analyses restricted to the bilateral amygdalae did not reveal any significant group x phase interactions. Increased activity was observed at trend-level in the right amygdala of subjects with PMDD compared to controls during the late-luteal phase (*p*_FWE_ < 0.10, TFCE, small volume corrected) (Figure S2), but not during the mid-follicular phase. No significant effects of menstrual cycle phase were seen in either group.

#### Group differences in associations between emotion-induced brain activity and ISO/ALLO levels

**Fig. 3:**
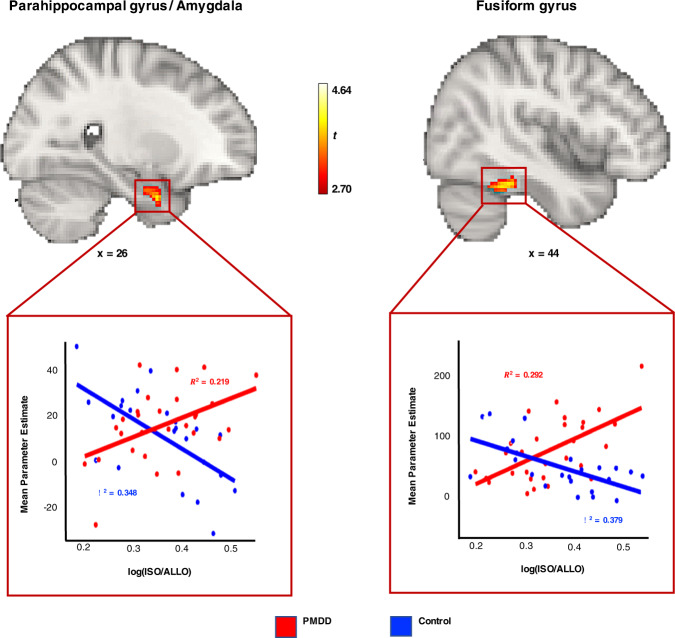
Group differences in associations between emotion-induced brain activity and log(ISO/ALLO) serum levels in subjects with PMDD (*N* = 29) and controls (*N* = 24) during the late-luteal phase of the menstrual cycle (*p*_FWE_ < 0.10, TFCE). *Upper:* Brain slices depicting clusters in which the interaction between brain activity during task [Faces>Shapes contrast] and log(ISO/ALLO) serum levels differed between groups (*p*_FWE_ < 0.10, TFCE). The cluster in the parahippocampal gyrus/amygdala region was significant at the *p*_FWE_ < 0.05 level, while the cluster in the fusiform gyrus was marginally significant with *p*_FWE_ = 0.06. The analysis was conducted voxel-wise within a mask combining brain regions showing increased task-related activation for both PMDD and controls. *Lower:* Corresponding scatter plots illustrating the relationships between mean parameter estimates extracted from significant clusters and serum ISO/ALLO levels. Simple regression lines were added for visualization. Subjects with PMDD exhibited positive associations between log(ISO/ALLO) levels and brain activity in the right parahippocampal gyrus/amygdala and right fusiform gyrus. Control subjects, on the other hand, showed significant negative associations between log(ISO/ALLO) and brain activity in both clusters. ALLO Allopregnanolone, FWE Family Wise Error correction, ISO isoallopregnanolone, PMDD Premenstrual Dysphoric Disorder, TFCE Threshold-Free Cluster Enhancement.

The association between task-related brain activity and ISO/ALLO serum levels was significantly different between subjects with PMDD and controls in the late-luteal phase in a cluster straddling the right parahippocampal gyrus (PHG) and right amygdala (*p*_FWE_ < 0.05, TFCE) and marginally significant in the right fusiform gyrus (FuG) (*p*_FWE_ < 0.10, TFCE) (Fig. 3, Table S6). In both clusters, there was a positive relationship between ISO/ALLO levels and brain activity in participants with PMDD, while the relationship between these two variables was negative in controls. No significant group difference was found in how serum levels of progesterone, estradiol, ALLO or ISO related to brain activity across the menstrual cycle.

#### Emotion-induced brain activity is associated with severity of anxiety symptoms in PMDD

**Fig. 4:**
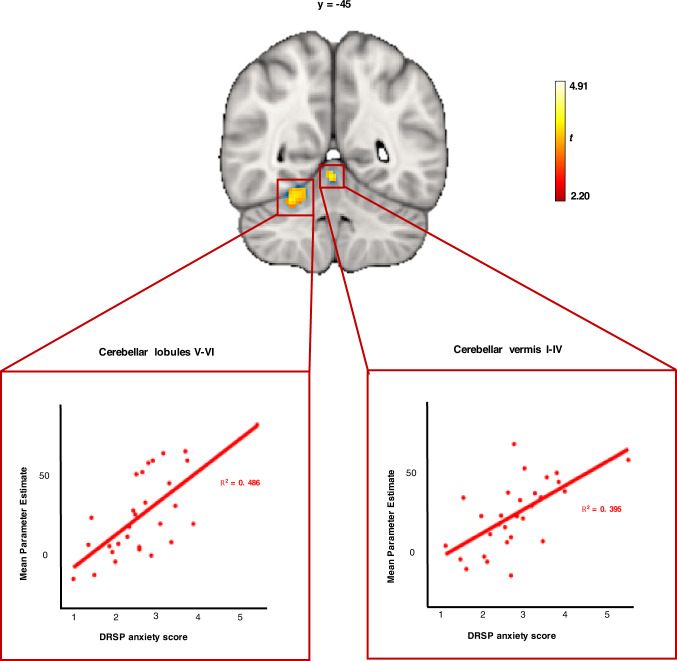
Associations between emotion-induced brain activity and anxiety scores in subjects with PMDD (*N* = 29) during the late-luteal phase of the menstrual cycle (*p*_FWE_ < 0.05, TFCE). *Upper:* Brain slice depicting clusters in which there was a significant linear relationship between brain activity during task [Faces>Shapes contrast] and anxiety scores (*p*_FWE_ < 0.05, TFCE). The analysis was conducted voxel-wise within a mask combining brain regions showing increased task-related activation in subjects with PMDD during the late-luteal phase. *Lower:* Corresponding scatter plots illustrating relationships between mean parameter estimates extracted from significant clusters and anxiety scores. Simple regression lines were added for visualization. DRSP Daily Record of Severity of Problems, FWE Family Wise Error correction, PMDD Premenstrual Dysphoric Disorder, TFCE Threshold-Free Cluster Enhancement.

Task-related brain activity within the right cerebellar lobules V-VI and the cerebellar vermis I-IV was significantly positively associated with DRSP anxiety scores in subjects with PMDD during the symptomatic late-luteal phase (*p*_FWE_ < 0.05, TFCE) (Fig. 4, Table S7). Brain activity was positively associated with total DRSP scores and emotional lability scores in the right cerebellar lobule V at trend-level (*p*_FWE_ < 0.10, TFCE). No significant relationships were found between task-related brain activity and scores on depression and irritability.

## Discussion

The present study found increased brain activity in response to emotional stimuli in individuals with PMDD in a number of brain regions hypothesized to be key components of emotion-processing brain networks, including the insula, as well as frontal and cingulate regions [35]. Notably, these differences were restricted to the late-luteal phase of the menstrual cycle, a period during which PMDD subjects experience a peak in symptom severity, and which is characterized by high levels of progesterone and its derivative neurosteroids ALLO and ISO. Furthermore, we found that the relative serum concentrations of ISO and ALLO (ISO/ALLO) were differently associated to emotion-induced activation of key regions involved in emotion processing (PHG and amygdala) and in the FuG. Lastly, emotion-induced activation of the cerebellum was positively associated to anxiety symptoms in subjects with PMDD during the late-luteal phase. Our study is the first to relate functional brain measures to GABA_A_-active neurosteroid levels in PMDD.

The brain regions found to functionally differ between groups in the late-luteal phase partly overlap with brain areas previously highlighted by task-based fMRI studies of PMDD patients, namely the insula, ACC, SFG, MFG, SMA, postcentral gyrus, precuneus, and cerebellum [30]. Among these, the insula and ACC constitute key hubs of emotion-processing networks [35]. Our findings are also in line with the localization of regions previously shown to functionally vary across the menstrual cycle, such as the insula, ACC, MFG, SFG, postcentral gyrus, and cerebellum [52], and which are thus probably particularly influenced by fluctuations of ovarian hormones. Regarding the direction of effects, some of our results were congruent with the existing neuroimaging literature on PMDD, such as greater activity in the insula, MFG, SFG, SMA and cerebellum [30, 31, 37, 53], while findings of increased activity in the ACC, precuneus, postcentral gyrus were not [30, 54, 55]. Additionally, the largest cluster of increased brain activity in PMDD subjects found in our study was located in the right PCC, a region not previously highlighted in the PMDD literature. The PCC is involved in emotion processing [56], but is a complex and functionally diverse region implicated in arousal and attentional focus as well as supporting internally directed thought and detecting changes in the environment [57]. Functional neuroimaging studies have shown altered PCC function and connectivity in a number of psychiatric disorders, including major depression [57]. Thus, our results partly corroborate existing neuroimaging studies of PMDD but certain inconsistencies in the localization and direction of effects motivate the need for replication in larger samples.

Significant correlations between serum progesterone and emotion-induced brain activity in the amygdala and dlPFC, as well as positive associations between serum estradiol and mPFC activity in individuals with PMDD have previously been reported [32, 37]. In the present study, neither progesterone nor estradiol levels showed significant relationships with emotion-induced brain activity. On the other hand, we found that higher ratios of ISO/ALLO were linked to higher activation of the right PHG/amygdala and right FuG in subjects with PMDD, while in controls negative relationships were observed. These findings are interesting for several reasons. Firstly, these brain regions have been shown to vary functionally across the menstrual cycle in subjects with PMDD [58], and have thus previously been implicated in the pathophysiology of PMDD. Secondly, the regions are implicated in emotion processing, whereby the amygdala is a key node of emotion processing networks [35], and the PHG is involved in the early appraisal and encoding of the emotional significance of stimuli during the automatic regulation of emotion [59]. The FuG, on the other hand, is involved in face processing [60], but is functionally influenced by the amygdala, as evidenced from a lesion study showing that increases in fusiform activation in response to emotional faces is impaired in subjects with amygdala damage [61]. Furthermore, the study showed that impaired FuG activity varied linearly with the degree of ipsilateral amygdala damage. The close functional relationship between these two brain regions might explain why the right FuG was found in conjunction with the right amygdala in our study. Thirdly, the amygdala seems to be particularly prone to neurosteroid influence as evidenced from a human post-mortem study, where ALLO was shown to accumulate at higher concentrations in the amygdala than in other brain areas [13], and an fMRI study, which showed that luteal phase levels of ALLO selectively increased amygdala activity in participants without premenstrual symptoms [62]. Fourthly, the 1:2 ratio of ISO/ALLO at which the lowest measures of fMRI activity were observed in controls reflects the relative dosage at which ISO’s antagonism of ALLO can be detected experimentally using measurements of saccadic eye velocity in subjects without premenstrual symptoms [23], and may thus be conjectured to have real physiological relevance. In sum, the altered relationship between ISO/ALLO levels and brain activity in key regions of emotion networks (amygdala, PHG) compared to controls may reflect a dysregulated modulation of GABAergic tone by neurosteroids in these areas in PMDD, which may help explain the mood symptoms associated with the disorder.

The present study found a positive association between anxiety symptoms and brain responses to emotional faces in the cerebellar lobules V-VI and anterior vermis in subjects with PMDD. These areas are part of the “emotional cerebellum” and are hypothesized to be involved in numerous aspects of emotion processing, including the perception, recognition, and evaluation of emotion, as well as its integration into behavior [63]. Structural, neurochemical and functional abnormalities have been observed in the cerebellum of patients with other psychiatric disorders, including bipolar and unipolar depression, which share aspects of PMDD symptomatology [63]. In PMDD, fMRI and positron emission tomography (PET) studies have observed greater activity in the emotional cerebellum relative to controls [64], and one study reported that increases in cerebellar activity from the follicular to the luteal phase was correlated to worsening of core mood symptoms [65]. In sum, there is some evidence to suggest that dysfunction in the emotional cerebellum plays a part in the neuro-pathophysiology of PMDD. This long-overlooked part of the brain warrants more attention in future research.

The present study has a number of strengths, amongst which are 1) its relatively large sample size in the context of neuroimaging studies of PMDD, 2) confirmation of menstrual cycle phase through cycle mapping and hormonal assessment, 3) minimization of order effects through the counterbalancing of menstrual cycle phase for the first scanning session, 4) usage of both ROI and whole-brain approaches to characterize brain function, 5) control for psychiatric history as a potential confounder, and 6) rigorous assessment of symptoms across the menstrual cycle in all participants (both PMDD patients and controls) using the prospective DRSP rating scale for at least two menstrual cycles prior to inclusion in the study. Despite its strengths, a number of limitations need to be considered. Firstly, although previous studies have shown that premenstrual symptom type and severity in an individual are relatively consistent and stable across menstrual cycles [66–68], our finding of positive associations between anxiety scores and emotion-induced brain activity in the cerebellum of subjects with PMDD should be viewed with caution as the symptom ratings used in the analysis were not collected during the actual scanning phase. In hindsight, it would have been preferable to continue symptom ratings through the scanning cycles. This oversight is a major limitation to the study; however, as ovulation was confirmed through a progesterone sample during scanning cycles, the luteal scans of PMDD subjects can reasonably be assumed to have been performed during symptomatic days. Secondly, it should be stressed that serum levels of ovarian hormones and neurosteroids do not necessarily reflect the precise hormonal milieu in local brain areas as steroids do not accumulate uniformly across the brain [13] and all necessary enzymes for de novo synthesis of ALLO and ISO and epimerization between the two steroids are present in neuronal and glial cells [26, 69]. Lastly, it has been argued that hormone ratios are fraught with interpretational difficulties, especially in cases where the biological mechanisms by which two hormones jointly influence an observed outcome are unclear [47]. Nonetheless, we found it apt to compound ISO and ALLO into a ratio variable due to the nature of the antagonistic relationship between the two neurosteroids, the lack of activity of ISO in the absence of ALLO, as well as the theoretical and empirical evidence that the relative concentration between the steroids is physiologically relevant for activity at the GABA_A_ receptor. Furthermore, we performed ALLO x group and ISO x group analyses in order to understand whether our results were driven by individual contributions by the original variables.

In conclusion, the present findings point to phase-specific differences in emotion-induced brain activity in individuals with PMDD. Furthermore, results suggest that brain activity in key emotion-processing regions may be differently influenced by physiological levels of the GABA_A_-active neurosteroids ISO and ALLO during the symptomatic luteal phase in PMDD. Further investigations at the brain and behavioral level of these potent modulators of GABAergic activity is merited.

## Supplementary information

Supplementary materials

Figure S1

Figure S2
