# Supplementary material for: Emotion-induced brain activation across the menstrual cycle in individuals with premenstrual dysphoric disorder and associations to serum levels of progesterone-derived neurosteroids
Source: Transl Psychiatry. 2023 Apr 14;13:124. doi: 10.1038/s41398-023-02424-3 (PMC10101953; doi:10.1038/s41398-023-02424-3)
Supplement: Supplementary file 1 — Supplementary materials [file 41398_2023_2424_MOESM1_ESM.pdf]

Supplementary materials

Tables

Table S1. Main effect of task. Peak activations for the Faces>Shapes contrast across groups and sessions.

k	MNI coordinates			*t*	Brain region
**14679**	22	-86	-8	36.9	Occipital fusiform gyrus R[1]
	12	-88	-6	30.0	Lingual gyrus R[1]
	-14	-100	2	29.6	Occipital pole L[1]
	18	-98	4	29.2	Occipital pole R[1]
	32	-86	-8	27.0	Lateral occipital cortex, inferior division R[1]
	44	-54	-18	25.8	Temporal occipital fusiform cortex R[1]
	-2	-90	-8	24.8	Lingual gyrus L[1]
	-34	-86	-8	22.9	Lateral occipital cortex, inferior division L[1]
**1663**	46	16	26	19.0	Inferior frontal gyrus R[3]
**1421**	-42	14	26	19.5	Inferior frontal gyrus L[3]
**736**	20	-30	0	27.8	Thalamus R
**479**	-6	14	50	16.6	Paracingulate gyrus L[2]
**349**	36	22	0	23.4	Insular cortex R[3]
**332**	-36	20	-2	21.7	Insular cortex L[3]
**316**	20	-4	-14	20.9	Amygdala R
**273**	-30	-56	42	16.1	Superior parietal lobule L[3]
**156**	-20	-4	-16	17.9	Amygdala L
**132**	-30	-66	-50	16.0	Cerebellum VIIb L
**116**	-38	-2	64	14.8	Middle frontal gyrus L[3]
**76**	-20	-36	-42	16.9	Cerebellum X L
**56**	30	-66	-48	12.9	Cerebellum VIIb R

Increases in task-related BOLD-signal were observed in bilateral amygdalae; in regions of the frontoparietal network: bilateral insular cortices, superior parietal lobule, bilateral inferior frontal gyri, and middle frontal gyrus; and in regions of the visual network: occipital fusiform gyrus, bilateral lingual gyri, lateral occipital cortices (local peak in the occipital face area) and temporo-occipital fusiform cortex (local peak in the fusiform face area). All coordinates are reported in MNI space. The locations of peak

activations were extracted from t statistic maps masked using a TFCE correction of $p_{FWE}<0.05$ and a grey matter probability map of 0.5. To achieve separation of fMRI clusters an additional threshold of $t>10.288$, $p<10^{-14}$, was set. Local maxima for the largest cluster (k=14679) are reported. Redundant peaks within the cluster are omitted. The Harvard-Oxford atlas was used for labelling. Clusters of k≥50 are reported. Yeo networks: [1]Visual network, [2]ventral attention network, [3]frontroparietal network. Abbreviations: BOLD, blood-oxygen level dependent; fMRI, functional Magnetic Resonance Imaging; FWE, Family Wise Error correction; *k,* cluster extent; L, left; MNI, Montreal Neurological Institute; R, right; TFCE, Threshold-Free Cluster-Extent.

Table S2. Main effect of task. Peak activations for the Faces>Shapes contrast for the PMDD group during the late-luteal phase.

k	MNI coordinates			*t*	Brain region
	30	-50	-30	14.1	Cerebellum VI R
**5315**	38	-62	-26	12.5	Cerebellum Crus I R
	-18	-48	-26	10.9	Cerebellum V L
**1269**	8	36	-2	10.3	Anterior cingulate gyrus R[1]
**342**	12	12	2	12.6	Caudate R
**92**	54	-44	-10	6.56	Middle temporal gyrus, temporo-occipital part R[2]
**91**	-8	14	0	10.9	Caudate L
**70**	20	-14	-30	5.78	Parahippocampal gyrus, anterior division R[3]
**50**	8	-48	-56	4.78	Cerebellum IX R
**45**	-4	60	8	4.91	Frontal pole L[1]
**36**	-6	-68	20	5.64	Precuneus L[1]
**27**	-54	-22	22	5.53	Postcentral gyrus L[4]
**24**	-54	-20	30	6.44	Postcentral gyrus L[5]
**20**	4	-80	20	6.07	Cuneal cortex R[6]
**17**	0	-46	-68	4.18	Brain stem

All coordinates are reported in MNI space. The locations of peak activations were extracted from *t* statistic maps masked using a TFCE correction of $p_{FWE}<0.05$ and a grey matter probability map of 0.5. Local maxima for the largest cluster (k=5315) are reported. Redundant peaks within the cluster are omitted. The Harvard-Oxford atlas was used for labelling. Clusters of k≥10 are reported. Yeo networks: [1]Default mode network, [2]dorsal attention network, [3]limbic network, [4]somatosensory network, [5]ventral attention network, [6]visual network. Abbreviations: FWE, Family Wise Error correction; *k,* cluster extent; L, left; MNI, Montreal Neurological Institute; R, right; TFCE, Threshold-Free Cluster-Extent.

Table S3. Emotional task performance in women with PMDD (N=29) and controls (N=27).

		PMDD (N=29) Mean (SE)	Control (N=27) Mean (SE)
**$\text{Accuracy}_{\text{Shapes}}$ (%)**	Mid-follicular	98.9 (0.44)	99.5 (0.23)
	Late-luteal	98.7 (0.33)	98.7 (0.37)
**$\text{Accuracy}_{\text{Faces}}$ (%)**	Mid-follicular	93.2 (1.08)	95.1 (0.81)
	Late-luteal	91.0 (1.43)	96.0 (1.00) *****
**$\text{RT}_{\text{Shapes}}$ (ms)**	Mid-follicular	1006.2 (42.7)	987.8 (33.2)
	Late-luteal	995.6 (42.9)	990.1 (33.4)
**RT_{Faces} (ms)**	Mid-follicular	2072.1 (86.2)	2213.7 (65.5)
	Late-luteal	2108.7 (64.2)	2295.5 (62.5) *****

Accuracy and reaction time (ms) measures are presented for each task condition in each phase, in women with PMDD and controls. No significant phase differences within groups were detected. However, women with PMDD showed lower accuracy and reaction times in the Faces condition compared with controls during the luteal phase. Group differences were assessed using paired and unpaired Student’s t-tests. *****Significant group difference at $p<0.05$. Abbreviations: ms, milliseconds; PMDD, premenstrual dysphoric disorder; SE, standard error.

Table S4. Group x Phase interaction effects on emotion-induced brain activity for the Faces > Shapes contrast in women with PMDD (N=29) and controls (N=27) (p_{FWE}<0.10).

k	MNI coordinates			*t*	p_{FWE}	Brain region
**Luteal-Follicular $_{Control}$ > Luteal-Follicular $_{PMDD}$**						
**31**	38	14	52	4.50	0.08	Middle frontal gyrus R[1]
**20**	10	26	58	4.55	0.09	Superior frontal gyrus R[2]
**Luteal-Follicular $_{Control}$ < Luteal-Follicular $_{PMDD}$**						
**N.S.**						

All coordinates are reported in MNI space. The locations of peak activations were extracted from *t* statistic maps masked using a TFCE correction of p_{FWE}<0.10 and a grey matter probability map of 0.5. The Harvard-Oxford atlas was used for labelling. Clusters of k≥10 are reported. Yeo networks: [1]Frontoparietal network, [2]default mode network. Abbreviations: FWE, Family Wise Error correction; k, cluster extent; MNI, Montreal Neurological Institute; N.S., Non-Significant; PMDD, Premenstrual Dysphoric Disorder; R, right; TFCE, Threshold-Free Cluster-Extent.

Table S5. Whole-brain group comparison between women with PMDD (N=29) and controls (N=27) of emotion-induced brain activity for the Faces > Shapes contrast in the late-luteal phase of the menstrual cycle.

k	**MNI coordinates**			***t***	**Brain region**
**PMDD > Control**					
**369**	10	-32	40	4.01	Posterior cingulate gyrus R[3]
**230**	-2	4	40	3.46	Anterior cingulate gyrus L[3]
**192**	30	28	36	3.60	Middle frontal gyrus R[4]
**181**	12	32	54	3.76	Superior frontal gyrus R[4]
**158**	-34	14	10	3.32	Insular cortex L[3]
**158**	10	-18	6	5.05	Thalamus R
**120**	34	18	12	3.78	Insular cortex R[3]
**71**	-10	-20	8	3.71	Thalamus L
**68**	-2	-8	64	3.16	Supplementary motor cortex L[1]
**67**	38	-8	6	3.48	Insular cortex R[1]
**61**	-47	-24	42	3.86	Postcentral gyrus L[1]
**57**	10	-6	72	3.06	Superior frontal gyrus R[1]
**51**	22	-6	58	3.40	Superior frontal gyrus R[2]
**41**	-16	-36	40	3.58	Posterior cingulate gyrus L[3]
**38**	48	-52	-36	3.86	Cerebellum Crus I R
**21**	26	4	4	2.85	Putamen R
**12**	-16	-12	22	3.25	Caudate L
**11**	-6	-46	52	2.90	Precuneus L[3]
**PMDD < Control**					
**N.S.**					

All coordinates are reported in MNI space. The locations of peak activations were extracted from *t* statistic maps masked using a TFCE correction of p_{FWE}<0.05 and a grey matter probability map of 0.5. The Harvard-Oxford atlas was used for labelling. Clusters of k≥10 are reported. Yeo networks: [1]Somatomotor network, [2]dorsal attention network, [3]ventral attention network, [4]default mode network. Abbreviations: FWE, Family Wise Error correction; k, cluster extent; L, left; MNI, Montreal Neurological Institute; N.S., Non-Significant; PMDD, Premenstrual Dysphoric Disorder; R, right; TFCE, Threshold-Free Cluster-Extent.

Table S6. Group x log(ISO/ALLO) levels interaction effects on emotion-induced brain activity for the Faces > Shapes contrast during the late-luteal phase in women with PMDD (N=29) and controls (N=24) (p_{FWE}<0.10).

k	**MNI coordinates**			***t***	***p*$_{FWE}$**	**Brain region**
**Slope PMDD > Slope Control**						
**217**	26	2	-30	4.64	**0.03**	Parahippocampal gyrus, anterior division R[1]
	32	0	-24	4.14	**0.04**	Amygdala R
	36	12	-44	3.57	0.07	Temporal pole R[1]
**101**	44	-38	-22	4.43	0.06	Temporal fusiform cortex, posterior division R[2]
**Slope Control > Slope PMDD**						
**N.S.**						

All coordinates are reported in MNI space. The locations of peak activations were extracted from *t* statistic maps masked using a TFCE correction of p_{FWE}<0.10 and a grey matter probability map of 0.5. Local maxima for the largest cluster (k=217) are reported. The Harvard-Oxford atlas was used for labelling. Clusters of k≥10 are reported. Yeo networks: [1]Limbic network, [2]dorsal attention network. Abbreviations: FWE, Family Wise Error correction; k, cluster extent; MNI, Montreal Neurological Institute; N.S., Non-Significant; PMDD, Premenstrual Dysphoric Disorder; R, right; TFCE, Threshold-Free Cluster-Extent.

Table S7. Associations between emotion-induced brain activity for the Faces > Shapes contrast and DRSP symptom scores in women with PMDD (N=29) during the late-luteal phase.

Direction of effect	*k*	MNI coordinates			*t*	Brain region
**Anxiety**						
**Positive**	**310**	20	-48	-20	4.91	Cerebellum V R
		26	-54	-22	4.78	Cerebellum VI R
**Positive**	**15**	2	-52	-8	4.28	Cerebellar Vermis I-IV R
**Total DRSP score**						
Positive	*62*	*22*	*-46*	*-24*	*3.81**	*Cerebellum V R*
**Emotional lability**						
Positive	*10*	*20*	*-46*	*-16*	*3.77**	*Cerebellum V R*
**Depression**						
	**N.S.**					
**Irritability**						
	**N.S.**					

The total DRSP score, and the scores of the four core PMDD symptoms, namely depression (items "depressed", "hopelessness" and "guilt"), anxiety (item "anxious"), affective lability (items "mood swings" and "easily hurt") and irritability (items "irritable" and "conflicts") were tested. Clusters indicating a positive relationship between anxiety scores and task-related brain activity were significant at $p_{FWE}<0.05$, TFCE, while clusters found for total DRSP scores and emotional lability scores (shown in italics) were only apparent when thresholding images at the $p_{FWE}<0.10$ (TFCE) level. All coordinates are reported in MNI space. The locations of peak activations were extracted from *t* statistic maps masked using a grey matter probability map of 0.5. Local maxima for the largest cluster (k=310) are reported. The Harvard-Oxford atlas was used for labelling. Clusters of k≥10 are reported. *Significant at trend-level ($p_{FWE}<0.10$, TFCE). Abbreviations: FWE, Family Wise Error correction; k, cluster extent; MNI, Montreal Neurological Institute; N.S., Non-Significant; PMDD, Premenstrual Dysphoric Disorder; R, right; TFCE, Threshold-Free Cluster-Extent.

Figure legends

Figure S1. Group x Phase interaction effects on emotion-induced functional reactivity in women with PMDD (N=29) and controls (N=27) (p_{FWE}<0.10, TFCE).

Upper: brain slices depicting clusters in which the interaction effect between group and phase for functional reactivity in the Faces>Shapes contrast was significant at trend-level (p_{FWE}< 0.10, TFCE). *Lower:* corresponding bar plots illustrating that the interaction effect is driven by women with PMDD showing increased functional reactivity in clusters from the mid-follicular to the late-luteal phase, while controls show significant phase-differences in the opposite direction, i.e. increased reactivity in the mid-follicular compared to the late-luteal phase. *Signifcant at p<0.05, **p<0.01, ***p<0.001. Error bars indicate 1 standard error of the mean. Abbreviations: FWE, Family Wise Error correction; PMDD, Premenstrual Dysphoric Disorder; TFCE, Threshold-Free Cluster Enhancement.

Figure S2. Group comparison between women with PMDD (N=29) and controls (N=27) of emotion-induced functional reactivity in the amygdala during the late-luteal phase of the menstrual cycle (p_{FWE}<0.10, TFCE).

A. Brain slice depicting the result of a voxel-wise comparison of functional reactivity for the Faces>Shapes contrast, small volume corrected for bilateral amygdalae. Women with PMDD exhibited trend-level increased reactivity in the right amygdala during the late-luteal phase, compared with controls (p_{FWE} < 0.10, TFCE). The cluster extent, *k,* of the cluster was 10, with a peak activation at MNI coordinate (22, -2, -24) with t = 2.69 and Cohen's d = 0.73. B. Bar chart showing the mean parameter estimates inside the amygdala cluster for each group. Error bars indicate one standard error of the mean. * p<0.05. Abbreviations: FWE, Family Wise Error correction; PMDD, Premenstrual Dysphoric Disorder; TFCE, Threshold-Free Cluster Enhancement.
