# Supplementary figures and images for: Emotion-induced brain activation across the menstrual cycle in individuals with premenstrual dysphoric disorder and associations to serum levels of progesterone-derived neurosteroids
Source: Transl Psychiatry. 2023 Apr 14;13:124. doi: 10.1038/s41398-023-02424-3 (PMC10101953; doi:10.1038/s41398-023-02424-3)

Superior frontal gyrus

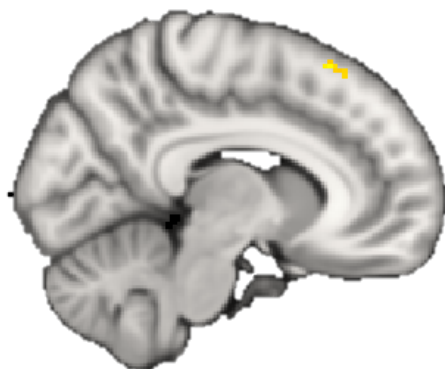

x = 10

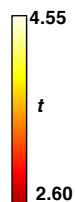

Middle frontal gyrus

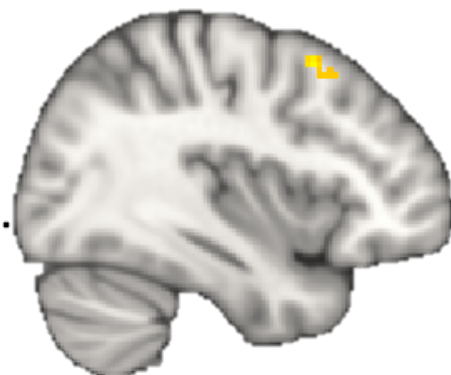

x = 38

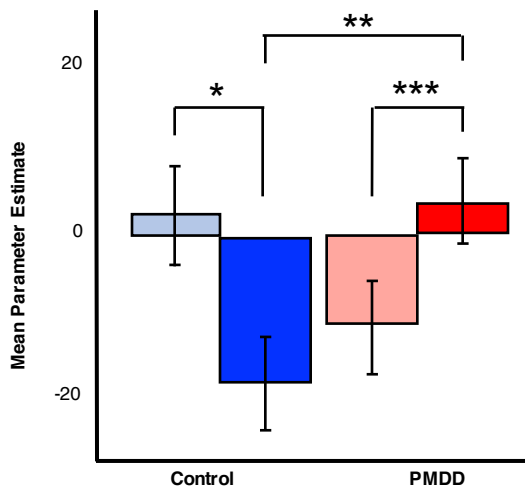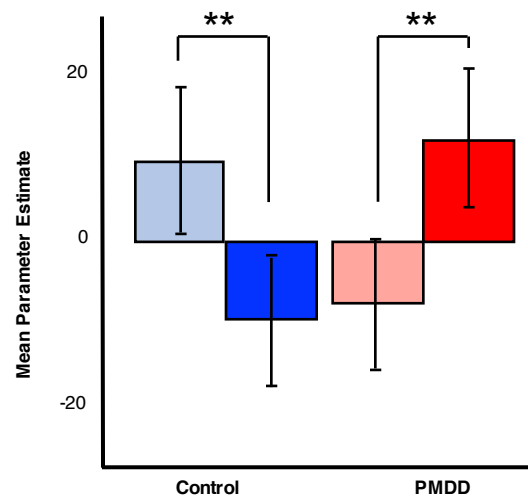

Follicular

Luteal

Supplement: Supplementary file 2 — Figure S1 [file 41398_2023_2424_MOESM2_ESM.pdf]

**A.**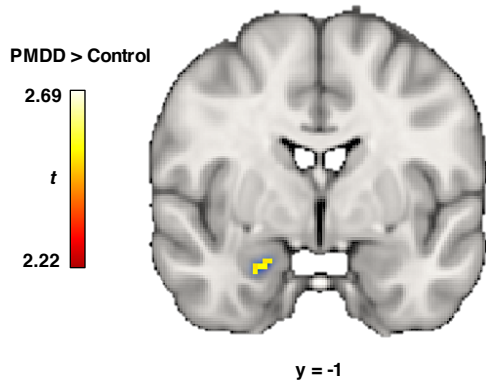**B.**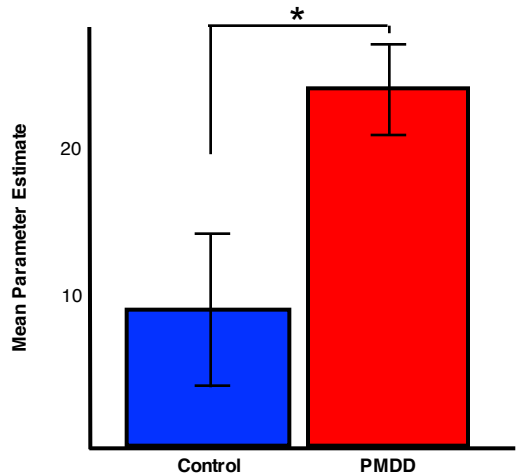

Supplement: Supplementary file 3 — Figure S2 [file 41398_2023_2424_MOESM3_ESM.pdf]
